# Activation of a Rhythmic Lower Limb Movement Pattern during the Use of a Multimodal Brain–Computer Interface: A Case Study of a Clinically Complete Spinal Cord Injury

**DOI:** 10.3390/life14030396

**Published:** 2024-03-16

**Authors:** Carla Pais-Vieira, José Gabriel Figueiredo, André Perrotta, Demétrio Matos, Mafalda Aguiar, Júlia Ramos, Márcia Gato, Tânia Poleri, Miguel Pais-Vieira

**Affiliations:** 1 Center for Interdisciplinary Research in Health (CIIS), Faculty of Health Sciences and Nursing, Portuguese Catholic University, 4169-005 Porto, Portugal; cvieira@ucp.pt; 2 Institute of Biomedicine (iBiMED), Department of Medical Sciences, University of Aveiro, 3810-193 Aveiro, Portugal; jose.gabriel.sf@ua.pt (J.G.F.); mafaldaaguiar@ua.pt (M.A.); julia.ramos@ubi.pt (J.R.); marciagato@ua.pt (M.G.); 3 Centre for Informatics and Systems of the University of Coimbra (CISUC), 3030-290 Coimbra, Portugal; avperrotta@dei.uc.pt; 4 Research Institute for Design, Media and Culture, Polytechnic Institute of Cávado and Ave, 4750-299 Barcelos, Portugal; dmatos@ipca.pt; 5 Department of Physics, University of Aveiro, 3810-193 Aveiro, Portugal; 6 Plano de Ação para o Apoio aos Deficientes Militares, 4405-029 Vila Nova de Gaia, Portugal; taniapoleri@gmail.com

**Keywords:** spinal cord injury, brain–computer interface, virtual reality, multimodal stimulation, central gait pattern generator

## Abstract

Brain–computer interfaces (BCIs) that integrate virtual reality with tactile feedback are increasingly relevant for neurorehabilitation in spinal cord injury (SCI). In our previous case study employing a BCI-based virtual reality neurorehabilitation protocol, a patient with complete T4 SCI experienced reduced pain and emergence of non-spastic lower limb movements after 10 sessions. However, it is still unclear whether these effects can be sustained, enhanced, and replicated, as well as the neural mechanisms that underlie them. The present report outlines the outcomes of extending the previous protocol with 24 more sessions (14 months, in total). Clinical, behavioral, and neurophysiological data were analyzed. The protocol maintained or reduced pain levels, increased self-reported quality of life, and was frequently associated with the appearance of non-spastic lower limb movements when the patient was engaged and not experiencing stressful events. Neural activity analysis revealed that changes in pain were encoded in the theta frequency band by the left frontal electrode F3. Examination of the lower limbs revealed alternating movements resembling a gait pattern. These results suggest that sustained use of this BCI protocol leads to enhanced quality of life, reduced and stable pain levels, and may result in the emergence of rhythmic patterns of lower limb muscle activity reminiscent of gait.

## 1. Introduction

Spinal cord injuries (SCIs) lead to a loss of quality of life [1] as well as changes in the functionality of multiple systems [2,3,4]. The use of brain–computer interfaces (BCIs), or brain–machine interfaces (BMIs), whereby brain activity is recorded and decoded in real time to control an avatar in an immersive virtual reality (VR) environment or a device, combined with tactile and thermal feedback has led to beneficial neuroplasticity effects [5,6,7,8,9,10,11,12]. Improvement in BCIs has been achieved both through improvements in the use of different classifiers [13,14,15,16,17], as well as through spinal cord stimulation [18], functional electrical stimulation [8], but also by improving immersiveness. We recently presented a BCI that innovated through the introduction of multiple complex virtual reality scenarios that allowed for simultaneous visual, auditory, and tactile feedback [10,19]. 

Despite these advances, the exact mechanism underlying the increase in neuroplasticity that occurs during neurorehabilitation programs with BCIs is not yet known [5,6,7,10,11,20,21]. Some authors suggest that there may be a relevant role of the sensation of “embodiment”, which is defined as the feeling that the controlled avatar’s body corresponds to the user’s own body [22,23,24]. 

The neuronal substrate that explains the phenomenon of embodiment is still under investigation. However, it appears to involve complex networks of primary and secondary regions dedicated to the representation and movement of the self [25,26,27]. Namely, it has been proposed that the experience of embodiment results from the complexity and organization of the stimuli received by the subject [28], which arise from the frontal and parietal regions [29,30]. Likewise, it is still unclear what effects these protocols have on pain [10]. The presence of neuropathic pain in SCI patients appears to be associated with cortico-thalamic dysfunction, which results in an increase in power of the delta and theta frequency bands and a reduction in the alpha band [19,31].

**Figure 1:**
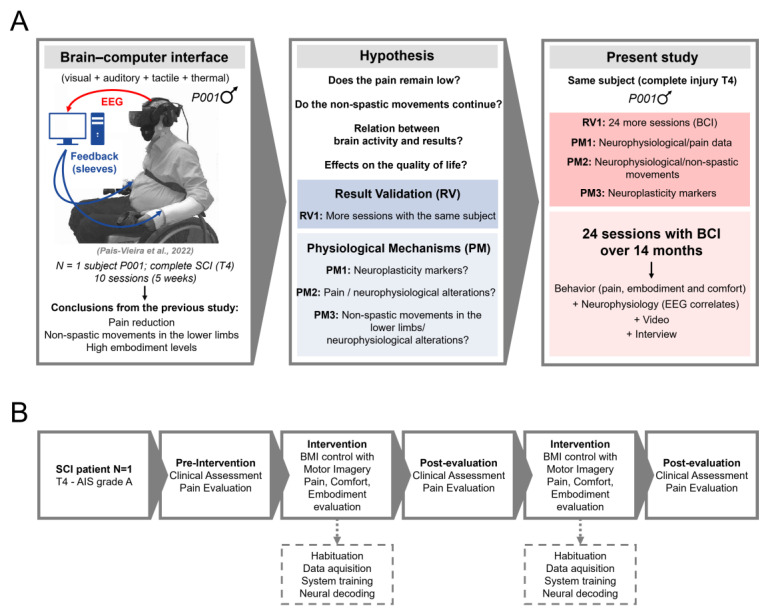
Implemented study design. (**A**) The left panel depicts the setup used, the patient’s characteristics, and main findings from a previous study [10]. The middle panel depicts the main questions and hypotheses tested in the present study. In the right panel, the characteristics and objectives of the study and the approaches taken to evaluate the patient’s condition throughout the testing period are presented. (**B**) Flow chart of the study. SCI: spinal cord injury; RV: result validation; PM: physiological mechanisms; BCI: brain–computer interface.

In a previous study, we presented the results of a VR-based BCI protocol with tactile and thermal feedback in a SCI patient with American Spinal Injury Association (ASIA) Impairment Scale (AIS) grade A (complete) at the fourth thoracic vertebra (T4) [10]. The implementation of this highly complex system with a previously nonexistent degree of realism led to a reduction in pain levels and, unexpectedly, the appearance of non-spastic movements of one or the other lower limb in two sessions. The patient reported that the use of the highly realistic BCI combining the tactile and thermal feedback with the hyper-realistic scenarios allowed him to relive his memory of walking as he did before the accident, which was a positive experience. In other words, the use of this system for a short period led to neuroplastic changes with relevant potential that, to the best of our knowledge, had not yet been described. Despite the importance of these previous results, the number of sessions was relatively short as it was a comfort assessment study, and thus, the main objective was to explore the user’s response to the equipment. As such, in the present study, we carried out a prolonged evaluation of the effects of implementing this protocol on the same patient in a block of 24 sessions which, together with the sessions previously carried out, took place over a period of 14 months (Figure 1A,B).

Our study hypotheses were as follows: Hypothesis (1): the variation in pain levels is related to the neuronal activity of the patient’s left frontal electrode [19,29,30,31,32]; Hypothesis (2): the appearance of non-spastic movements of the lower limbs is dependent on the use of the BCI and can be induced in a controlled manner; and Hypothesis (3): non-spastic movements of the lower limbs present a repetitive and nonrandom pattern characterized by a specific neuronal activity. 

The first hypothesis concerns the possibility of predicting the patient’s self-reported pain solely using neuronal activity. This hypothesis is based on previous studies where it was possible to predict neuropathic pain in patients with SCI solely through electroencephalography (EEG) signals [19,31,32], as well as through the combination of descriptions of neuronal activity associated with embodiment [29,30]. The second hypothesis is related to the apparent periodicity of lower limb movements observed in two sessions of the previous study. If there are movements of the lower limbs, and if they have a clear periodicity, then these movements may be an indicator that the BCI combining VR with tactile and thermal feedback is activating central mechanisms at the spinal and/or cerebral level [5,7]. The third hypothesis is related to the neurophysiological substrate of lower limb movements, namely the possibility of this mechanism being medullary or cortical. Central pattern generators at the spinal cord level in cats were identified that generated highly organized patterns of lower limb activity resembling walking, independent of cortical activation, which were also proposed in humans [33,34,35]. However, if neuronal correlates of non-spastic movements are found in the EEG signal, this would suggest the presence of a cortical pattern generation mechanism and, potentially, the existence of some spared medullary fibers, even though this patient was classified as an AIS grade A [36].

## 2. Materials and Methods

### 2.1. Patient, Ethics Approval, and Consent to Participate

This case study was conducted with the approval of the Hospital da Senhora da Oliveira-Guimarães (HSOG) ethics committee (Ethical approval #15/2020). The patient provided informed consent before enrolling in this study. He was 52 years old, male and had been diagnosed with an SCI classified as AIS grade A at the T4 level. This injury was stabilized (32 years). This patient was identified as P001. Throughout this study, the term “assessment” is used to refer to any type of evaluation conducted as part of the study. This may encompass interviews, BCI sessions, questionnaire administrations, or AIS grade evaluations. A “session” indicates instances when the patient underwent testing in the BCI setup, which necessarily included the evaluation of multiple variables such as pain and embodiment. On the other hand, the term “clinical assessment” (a1, a2, and a3) is used specifically to describe the clinical evaluation of the AIS grade by a physiatrist or neurologist.

### 2.2. VR and BCI Systems

The system used in this study has been previously described [10,37]. This BCI consists of a VR headset (for visual and auditory stimuli), two manual controls (used during the habituation phase) (HCT VIVE Pro Eye, New Taipei City, Taiwan), and a pair of thermo-tactile sleeves (Figure 1A). Thermo-tactile sleeves can deliver different patterns of thermal and tactile stimuli. These patterns can be programmed to match the type of terrain where the virtual soles of the avatar are stepping in (e.g., grass, beach sand, stone, etc.). The sleeves had independent mechanisms for temperature and tactile feedback. Tactile feedback was delivered to the user’s forearms through vibrating motors, reflecting the avatar’s movements. Thermal feedback was provided through aluminum plates, maintaining temperatures between 17 °C and 38 °C, which exchanged heat with the user’s forearm skin. Each scenario was associated with a specific temperature. In scenarios involving different terrain transitions, different temperatures were used for each terrain type. Water scenarios utilized lower temperatures (<25 °C). Lastly, neural activity was recorded using an EEG recording system (16 channels, V-Amp, actiCAP; Brain Products GmbH, Gilching, Germany) running on OpenViBE (v2.2.0) [13]. The synchronization and command software for the entire system was running in Max (Cycling ‘74, v8.0.8, San Francisco, CA, USA), and the VR scenarios were run in Unity (Unity Technologies, v2019.3, San Francisco, CA, USA).

### 2.3. Intervention and Sessions

The intervention had two parts (Figure 1A,B). The first and “Initial” part consisted of two weekly sessions, for 10 sessions (S1–S10). The second and “Final” part consisted of 24 weekly sessions (S11–S34) followed by a clinical assessment, in 14 months of testing, as shown in Figure 1. Sessions were conducted as previously described [10,37]. Briefly, each session was carried out by a group of 1–4 researchers and lasted between 70 and 90 min, which included a comfort assessment (10 min) and interaction with the VR environment (20–25 min: habituation, data acquisition, and real-time decoding; described in detail below). The remaining time was for positioning, calibration, and placement of the equipment, confirming appropriate feedback from each different component of the multimodal feedback, checking EEG signal and impedance, as well as removing the gear and cleaning the EEG gel. During the sessions, notes were collected—in addition to the questionnaires listed in the next section—regarding comfort and embodiment experiences reported by the patient, for later analysis. In three sessions, the patients’ legs were exposed, and video recordings of the lower limbs were performed using a cell phone camera. Two of these videos presented enough quality to be later analyzed frame-by-frame to identify potential muscle movements. Reference points (e.g., wheelchair) were used to determine muscle movements.

### 2.4. Evaluation of Embodiment, Comfort, Pain, and AIS Scale

The assessment of comfort related to motor imagery experiences of the lower limbs was carried out at the end of each session using an adaption of the Embodiment Questionnaire [10,38], the Faces Pain Scale [39], the Verbal Pain Intensity Scale [40], the Visual Analogue Scale (VAS) [41], with the side effects of VR controlled using the Simulator Sickness Questionnaire [42]. We aimed to evaluate the comfort associated with the lower limb embodiment experiences generated by using the system while ensuring the absence of pain or VR-associated side effects. In addition, since the system integrated a sleeve capable of generating tactile and thermal stimuli, the patient was also asked about its placement, comfort with the different types of stimulation provided, as well as the sleeve’s contribution to the overall immersive experience during the task. The AIS grade was evaluated at three different time points to check for potential improvements in sensory or motor functions [36]. Besides the evaluation during motor imagery sessions, the three different pain questionnaires were also applied immediately after clinical assessments.

Paired with the regular experiment time, a 5–10-min break was included per session. Here, the patient was encouraged to report his experiences either during the session, regarding the way the study was being conducted, or even potential changes or situations that had occurred outside the context of the session (e.g., changes in the frequency or intensity of pain episodes, appearance of other health problems, professional events that generated stress, etc.).

### 2.5. VR Environment

Sixteen virtual scenarios involving different types of landscapes were available. The patient could be exposed to multiple terrain combinations that resulted in different visual, auditory, tactile, and thermal stimuli within the same scenario. The patient could choose the scenario he wanted to go through in each session, as well as the general presentation of the avatar (e.g., the avatar’s skin tone and characteristics, clothing, shoes, etc., were kept as close as possible to the patient’s characteristics). Although the avatar was in a first-person perspective, the movement of the hands or head allowed the patient to observe parts of the avatar’s body within the VR environment. The aim of this correlation between the movements of the avatar and the patient’s hands was to increase the feeling of embodiment towards the avatar (i.e., to increase the feeling that “my body and the avatar’s are the same”).

### 2.6. Interaction with the VR Environment

Sessions began after system calibration. The interaction with the VR environment consisted of three different phases: (a) habituation; (b) EEG baseline with acquisition of neuronal data for training the neuronal network; and (c) testing real-time decoding of neural activity without avatar control.

*Habituation.* During this phase, the patient chose a scenario and interacted with it by making the avatar take a step, using the hand controllers. This period allowed any necessary adjustments to the VR environment, wheelchair position, or feedback sleeves to be identified. At this stage, the Simulator Sickness Questionnaire [42,43] was applied to identify potential side effects generated by the interaction with the VR environment.

*EEG baseline and neural data acquisition.* During the baseline period, 30 s of neural activity was recorded with the eyes open within the scenario that would then be used. This period made it possible to establish a clear separation between the habituation phase (more technical and to ensure the proper functioning of the equipment) and the remaining phases (testing period), where the patient needed to be calm and concentrated.

In the neural data acquisition, as well as in the following phase, colored cues appeared in the scenario to indicate to “Walk” (green cue), or “Stay still” (red cue), always preceded by a grey cue indicating that a trial would start soon. Of a total of 40 trials performed in each acquisition period, 20 trials were associated with “Walk” and 20 trials with “Stay still”. At the beginning of this phase, the patient was instructed that, when a green cue appeared, he should imagine that he was taking a step with his lower limb and, when a red cue appeared, he should imagine that his lower limbs were not moving. After acquiring neural data, the classifier was trained, and the resultant confusion matrix was analyzed. If the success rate was equal to or above 70%, the training would continue to the next phase. Otherwise, data acquisition and network training would be repeated.

*Real-time decoding of neural activity.* Neural activity was decoded in real time using an adaptation of the motor imagery decoding algorithm of the OpenViBE (v2.2.0) [13] software. This information was then used to control the rest of the BCI. The present study had two parts that differed in this last phase. The first part was without BCI performance feedback and included sessions S1–S10. Regardless of the patient’s neural activity, the avatar moved whenever a green cue appeared (with a delay of 2–3 s), and it remained in the same location when a red cue appeared. This was to remove the interference of negative reinforcement from the potential inability to generate motor imagery commands. In this way, only positive reinforcements were used. The result would then be presented to him at the end of the session so that he could have feedback on the strategies he was using to generate motor imagery commands. Meanwhile, the second part (sessions S11–S34), included immediate BCI performance feedback, namely through a high-pitch sound for correct neural decoding and low-pitch sound for incorrect neural decoding. In sum, in the first 10 sessions, the patient was not immediately informed of the results of the neural decoding during the trials (i.e., only at the end of the session), while in the second part, he received auditory feedback about the outcome of the neural decoding. In both cases the avatar moved after a green cue was presented and neural activity was evaluated (independently of the decoding being correct or incorrect).

### 2.7. Electroencephalography Recordings and Analysis

Electroencephalography data were recorded from 16 electrode channels Fp1, Fp2, F3, Fz, F4, C3, Cz, C4, T3, T4, Tp10, P2, Pz, P4, O1, and O2, placed following the 10–20 electrode system, at a sampling rate of 1000 Hz. The equipment used was V-Amp and actiCAP from Brain Products GmbH in Gilching, Germany, along with Brain Vision Recorder (version 2.1.0, Brain Products, Gilching, Germany). Subsequent offline data analysis was conducted with Brain Vision Analyzer (version 2.2.1, Brain Products, Gilching, Germany), as well as MATLAB (Mathworks, R2023a, Natick, MA, USA). During preprocessing, all channels were utilized as a reference for re-referencing. A 50 Hz notch filter was applied, and a zero-phase shift, 4th order Butterworth filter was employed with a low cutoff of 0.5 Hz and a high cutoff of 70 Hz, utilizing a time constant of 0.3183. Ocular artifacts were corrected using the Gratton and Coles algorithm, integrated into Visual Analyzer. Neural data analysis encompassed the entire acquisition phase of the session. A fast Fourier transform was subsequently applied with a resolution of 0.5 Hz. Power spectral analysis was performed within specific frequency bands: delta (0.5–4.5 Hz), theta (4.5–8.5 Hz), and alpha (8.5–13.5 Hz). Z scores were calculated, and comparisons were made between different groups of sessions using the permutation test (exact) as implemented in MATLAB.

### 2.8. Statistical Analysis

Results are presented as mean and standard deviation (Mean ± SD). Quantitative results of questionnaires are expressed in arbitrary units (a.u.). For hypothesis tests, a significance level of 5% (α = 0.05) was employed. Changes in electrophysiological signal measured at each electrode over time were assessed using the exact permutation test (utilizing the “permutation Test” function in MATLAB) to compare power within each frequency band between the different initial and final periods. To examine associations between pain levels and neural activity, Spearman’s correlation was used, with the Benjamini and Hochberg correction applied when multiple comparisons were made. It is important to note that data from pain scales encompass various aspects of pain and do not necessarily represent a linear scale [44]. These data are presented on the same graph to facilitate visualization of variations in multiple measurements across the study in each assessment. For clarity, the data from the verbal scale were converted into a numeric scale and are presented in the figures. When multiple sessions/evaluations are combined the average of this numeric scale is presented.

## 3. Results

The results are presented starting with a brief description of the characteristics of the sessions, followed by the general neurophysiological aspects that changed during the implementation of this protocol. Subsequently, the assessment of pain and its neural correlates are described, and then the results related to the induction of lower limb movement patterns when using the BCI are presented. Finally, the findings associated with reports related to the occurrence of thermal sensations and patient engagement in the neurorehabilitation protocol are described. As the study progressed, it was necessary to make decisions regarding additional variables to be collected or manipulated. Therefore, partial elements of discussion are included in the results to facilitate comprehension of the rationale for specific measurements.

### 3.1. Number of Clinical Assessments

The present study included 37 evaluations of patient P001. Among these, 12 evaluations pertained to the results previously presented [10] (including baseline clinical assessment-a1, 10 sessions, and follow-up clinical assessment-a2). The remaining 25 evaluations were associated with 24 additional sessions conducted over 11 months, alongside a clinical assessment (a3). In total, this intervention spanned 14 months. It is important to note that the inclusion of the results from the previous study in the present one was essential to establish a temporal pattern for comparing clinical variables and their neurophysiological correlates.

### 3.2. Electrophysiological Changes Suggestive of Neuroplasticity

**Figure 2:**
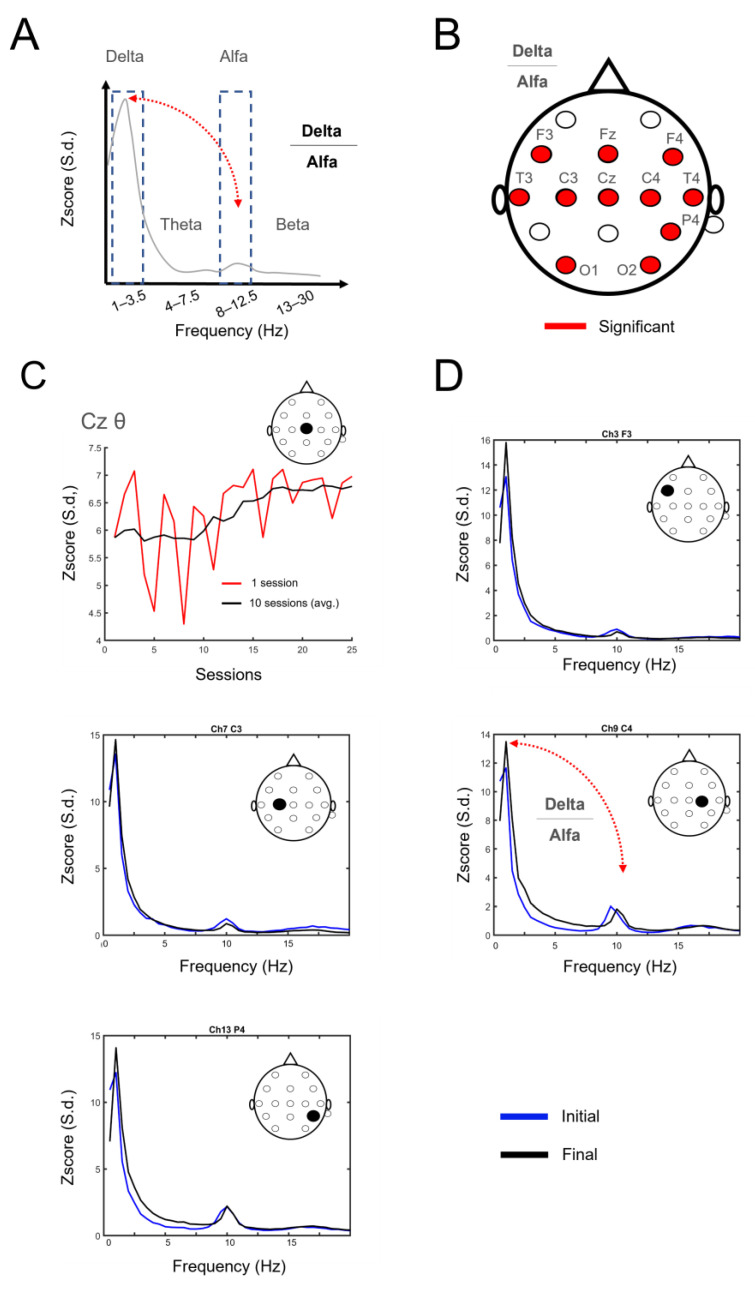
Decoding of pain levels from neural activity. (**A**) Neural frequency bands as a function of power. Example of power spectrum delta/alpha frequency band calculation. (**B**) Network of electrodes where changes in delta/alpha were significant (red fill, *p* < 0.05). From the 16 used electrodes, this outcome was observed in frontal (F3, Fz, F4), temporal (T3, T4), central (C3, Cz, C4), parietal (P4), and occipital (O1, O2) regions. (**C**) Example of changes in theta band power in electrode Cz throughout the final sessions (S11–S34). An overall reduction in power variability (red line, measurement) appears while an increase in power occurs over time (black line). (**D**) An example of the analysis of this ratio in electrodes F3, C3, C4, and P4 in the initial (blue line) and the final (black line) sessions.

In all the sessions, a success rate above 70% during the BCI classifier training phase was achieved. Thus, there were no restarts of the classifier training during this experiment. A comparison of the electrophysiological signals between the first (initial) and second sessions (final; see Figure 2A–D) revealed that implementing this protocol resulted in changes in signal power in multiple frequency bands. As shown in Figure 2B–D, these differences were more pronounced in the lower frequency bands (delta, theta, and alpha). In Figure 2D, the power spectra of four electrodes positioned in the frontal (F3), central (C3, C4), and parietal (P4) regions illustrate the variations in signal power within the different frequency bands at the study’s onset (blue) and conclusion (black). An examination of the ratio between the power of the delta frequency band and the alpha frequency band (i.e., delta/alpha) revealed the existence of a widespread network of electrodes with statistically significant changes in the frontal (*p*-values adjusted for permutation tests with Benjamini and Hochberg’s correction; F3: *p* = 0.0213, Fz: *p* = 0.0378; F4: *p* = 0.0378), temporal (T3: *p* = 0.0378, T4: *p* = 0.0378), central (C3: *p* = 0.0378, Cz: *p* = 0.0378, C4: *p* = 0.0160), parietal (P4: *p* = 0.0160), and occipital (O1: *p* = 0.0378, O2: *p* = 0.0378) regions (Figure 2B). Figure 2C illustrates the evolution of power in the theta band recorded at the central electrode Cz across various sessions. In the graph, the red line represents the theta band power for each session, while the black line represents the average of multiple sessions. Notably, there was an overall reduction in power variability, indicated by the diminishing oscillation of the red line as the number of sessions increased. Concurrently, there was an increase in power over time, as depicted by the black line. These combined changes suggest that the implementation of this neurorehabilitation protocol induced alterations in electrophysiological signals across multiple regions, possibly indicative of a form of neuroplasticity in the patient.

### 3.3. Sustained Reduction of Pain Levels

**Figure 3:**
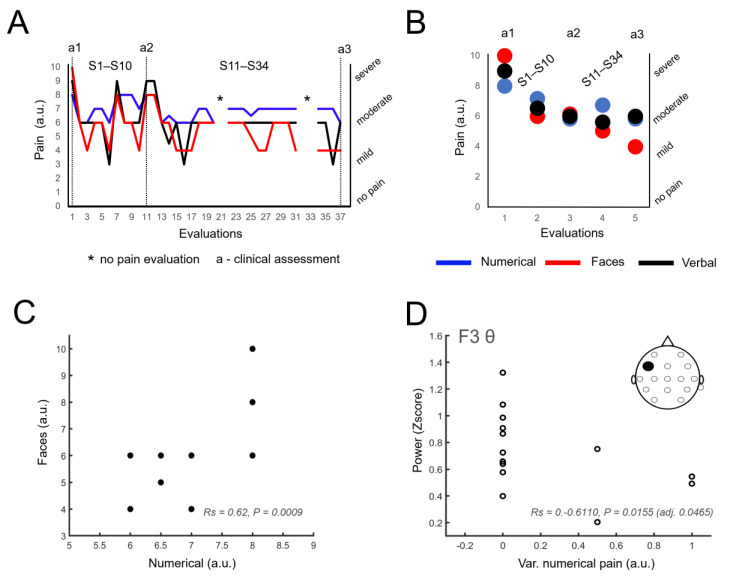
Evolution of pain levels on the different scales. (**A**,**B**) show (at each session and in intervals, respectively) a decrease in pain levels, obtained from the three different self-reported pain scales (numerical Visual Analogue Scale, Faces Pain Scale, and Verbal Pain Intensity Scale), during this study. (**C**) Observed correlation between numerical and faces pain scales from the reported values (Rs = 0.62, *p* = 0.0009). Note that some sessions have superimposed values and therefore, some circles cannot be observed in the panel. (**D**) Higher theta band power is correlated with a lower variability of the self-reported score of the VAS.

The analysis of the three different self-reported pain scales (numeric-VAS, faces scale and verbal scale) revealed a reduction in pain levels from the beginning of the intervention to its conclusion (Figure 3A,B). At the initial clinical assessment (labeled as “a1” in Figure 3A), the patient reported a score of 10 on the faces scale (indicated in red), a score of 8 on the numerical pain scale (in blue) and described the pain as “severe pain” on the verbal pain scale (in black). While variations were observed in all three scales, by the end of the first part of the intervention (labeled as “a2”, corresponding to clinical assessment 2 as presented in the previous study) [10], there had already been a reduction in pain levels that varied between two (numeric scale) and four points (faces scale). In the case of the verbal scale, the reduction was one level, transitioning from “severe” to “moderate”.

The continuity of the intervention over the subsequent 11 months (S11–S34) demonstrated a maintenance or reduction in these pain levels. Specifically, there was a reduction of two points on the numeric scale (from 8 to 6) and a reduction of six points on the faces scale (from 10 to 4) throughout the entire intervention. Self-reports of pain on the verbal scale showed a transition from “severe pain” in the first assessment to “moderate pain” in the second and third assessments, with “mild pain” reported in three sessions. Over the entire intervention period, there were only three reports of “severe pain”, occurring during the first assessment prior to commencing the intervention (“a1”) and two during the initial sessions (S1–S10). Notably, no reports of “severe pain” during the 11-month period encompassing the second phase of this study took place.

In addition to observing a trend toward reduced pain levels in the various assessments, it was also noted that the values reported on the numeric and faces pain scales showed a strong correlation (Rs = 0.62, *p* = 0.0009), as depicted in Figure 3C. This finding was further utilized to investigate the neural correlates of pain.

Taken together, the behavioral results from the pain scales lend support to the idea that the intervention contributed to a decrease in pain across the different scales, and these values either remained stable or decreased by the end of the intervention.

### 3.4. Identification of Neural Correlates of Pain

As pain values exhibited a tendency to decrease and considering that neural activity also displayed changes over time, a correlation analysis was conducted between pain values (VAS) and the power of the delta, theta, and alpha bands in the F3 channel [19,31,32]. The analysis revealed that increases in theta power were predictive of changes in pain levels (delta: Rs = −0.4791, *p* = 0.0708, adjusted *p* = 0.0708, n.s.; theta: Rs = −0.6110, *p* = 0.0155, adjusted *p* = 0.0465; alpha: Rs = −0.5190, *p* = 0.0474, adjusted *p* = 0.0708 n.s.) [19] (Figure 3D). These findings support the notion that neural activity recorded at the F3 frontal electrode encodes changes in patient-reported pain levels.

### 3.5. Other Information Related to Pain

During the sessions, the patient was asked about the pain levels present on the day of the session and about the pain levels on the remaining days of the week. Throughout the entire intervention, the patient never reported an increase in pain levels due to the intervention, either immediately after the session or overnight. This suggests that the effects induced by multimodal stimulation did not result in temporary or permanent increases in pain levels.

Finally, throughout the study period, there were no alterations to the dosage or type of prescribed pain control or other medications that could account for the changes found in electrophysiological signals [32].

### 3.6. Induction of Lower Limb Movement Patterns

In our previous study, we found the occurrence of non-spastic macroscopic movements in the lower limbs in the last two sessions, and this classification was independently confirmed by two specialists (physiatrist and neurologist). These movements were nonconscious, manifesting during the session while the patient wore a VR headset, and could appear in either limb. While spastic movements are associated with sudden uncontrolled action of one or more groups of muscles, or an excessive reflex response upon light touch stimulation [35,45], the activity observed here was coordinated. It started with dorsiflexion of the foot, which was followed by flexion of the leg and in some sessions, also of the hip. These movements were not correlated to the motor imagery. Instead, a rhythmic lower limb movement pattern appeared (typically in one limb) and was maintained for several minutes independently of the commands generated for the BCI during the task (i.e., “Walk”, “Stay still”). In the current study, we were able to verify the emergence of lower limb movements in 15 (62.5%) out of the 24 additional sessions (Final sessions). No changes were observed in the AIS classification, or in the motor and sensory scores.

A comparison of neurophysiological signals between sessions with lower limb movements and sessions without movements did not reveal any differences in power for electrodes C3 and C4 in the delta band (C3: without movements: 5.57 ± 1.37; with movements: 6.42 ± 0.97, *p* = 0.2727, n.s.; C4: 5.68 ± 1.01, without movements: 6.17 ± 0.57; *p* = 0.3497, n.s.).

To provide a more detailed description of these movements (from now on referred to as macro-movements), a video-based motion analysis was performed in two sessions (S31 and S32). In these sessions, both lower limbs were partially exposed to allow for better observation and control of variables. These video recordings were performed as a way of comparing the activity of the nonmoving limb (i.e., the “control” limb) with that of the moving limb. Unexpectedly, when observing the “control” limb without the clothes, small muscle movements could be observed (from now on referred to as micro-movements).

**Figure 4:**
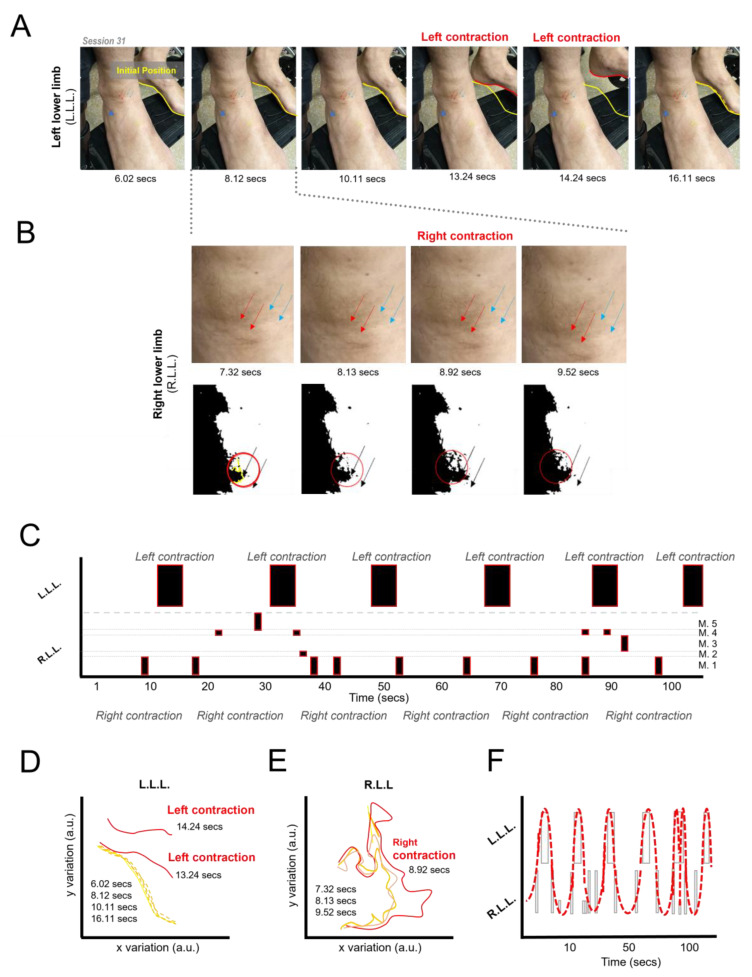
Alternating lower limb movement pattern. (**A**) Right (R.L.L) and left lower limb (L.L.L.) positions during an interval of approximately 10 s. The yellow line indicates the starting position, the orange lines indicate intermediate positions, and the red lines indicate the largest difference in position. (**B**) Top row: magnification of R.L.L frames. Bottom row: same video frames present in top row, with a black/white filter to show the right lower limb (R.L.L) micro-movements (red and blue arrows point to the observed muscle micro-contractions, red circle highlights the identified change) in video frame and graphical representation (light threshold mask, summary), respectively. Initial position (yellow line, 6 s), the position during a movement (red line, 13–14 s) and the return to the initial position (dashed yellow line, 16 s). Also, refer to Supplementary Materials Video S1. (**C**) Alternating pattern of muscle movements between the two lower limbs through time. (**D**) and (**E**) show, respectively, the graphical representation of the L.L.L. movements, as described above, and the graphical representation of the light threshold masks’ variation over time regarding the micro-movements seen in the right (R.L.L.; opposite) lower limb. Regarding (**E**), initial position (yellow line, 7 s), first position during a movement (orange line, 8 s), second position during movement (red line, 9 s), and end of the movement (pink line, 9.5 s). (**F**) Summary and graphical representation of the pattern with a line connecting the intervals between each lower limb movement (red dashed line).

The analysis of session S31 is depicted in Figure 4A–F. In Figure 4A, a sequence of images illustrates the initial position (yellow line, 6 s), the position during a movement of the left lower limb (red line, 13–14 s), and the return to the initial location (dashed yellow line, 16 s). These differences are summarized in Figure 4D. During these additional measurements, it was discovered that the lower limb intended to serve as a control also exhibited muscle movements, albeit smaller in amplitude. These movements were only observable when the lower limb was exposed, which explains why they had not been identified previously (Figure 4B,C,E, and Supplementary Video S1). The recording of these micro-movements was then compared with the macro-movements of the opposite limb and represented on a timeline. In Figure 4C, the activation pattern of different putative muscles (M1–5 representing distinct muscle areas) is presented. These activations primarily occurred during periods when there was no activity in the opposite limb, with a frequency ranging from 0.05 to 0.125 Hz.

This pattern of alternating movements between the lower limbs, resembling a gait pattern automatically triggered by the implementation of the BCI protocol, did not occur in all sessions (Table 1, see “Limb Mov.” column). Occasionally, macroscopic movements appeared in both lower limbs, but only for a brief period, after which the activity remained present in a single lower limb (e.g., session 21).

A more detailed analysis of the patient’s reports and the presence of movements in the sessions revealed that, as a rule, there was some form of lower limb movement in all sessions of the second part of the intervention (“Final”), unless there were technical problems or reports of stress-inducing events by the patient (Table 1, see “Stress Column”). As presented in Table 1, lower limb macro-movements were seldom observed in sessions affected by any type of event that impaired the patient’s concentration, such as arriving late, work-related problems, or technical problems (hardware/software) (S15).

**Figure 5:**
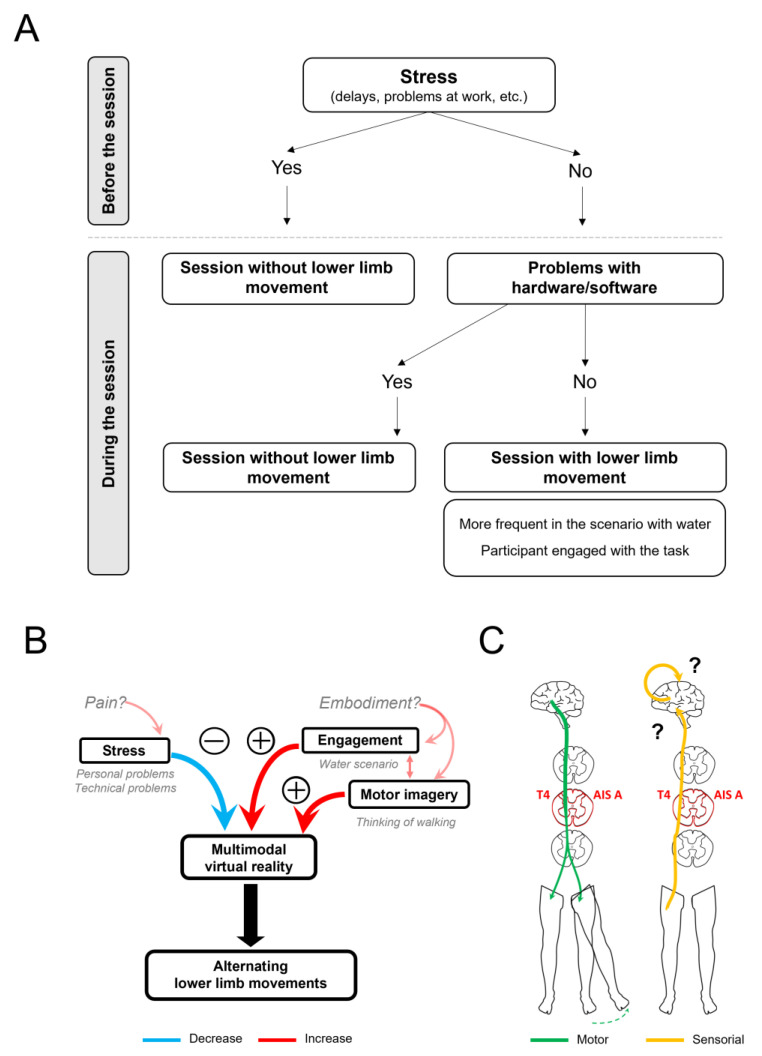
Factors that may influence the emergence of lower limb movements. (**A**) Decision tree proposing that lower limb movements are affected by stress-generating events. (**B**) Variables that may influence the appearance of the alternating pattern of lower limb movements seen during the brain–computer interface (BCI) sessions using multimodal (visual, auditive, thermal, and tactile feedback) virtual reality (VR). The red color indicates a positive modulation while the blue color indicates a negative modulation. (**C**) Potential efferent and afferent pathways activated with the use of this VR-based BCI protocol.

These findings suggest that factors leading to stress (software/hardware problems, anxiety) and reduced engagement (e.g., concerns about upcoming travel) inhibited the occurrence of lower limb movements. In Figure 5A, a decision tree illustrates the apparent effects of stress and engagement on this BCI protocol, and in Figure 5B,C are illustrated the factors and pathways that may be involved in the appearance of lower limb movements and the sensation of cold feet.

### 3.7. Control Manipulations Related to the Generation of Movements of the Lower Limbs

To gain a deeper understanding of the origins of the observed movement patterns, additional manipulations were introduced in the experimental environment. Specifically, we manipulated variables aimed to reduce stress and/or enhance patient engagement whenever feasible.

During a session (S15) when the patient was slightly anxious due to tardiness (S15), he did not exhibit any lower limb movements. However, later in the same session, he was placed in the water scenario and instructed to “think about walking” (engage in motor imagery). At this point, left lower limb movements emerged, and he reported “feeling his feet cold” (also refer to “*Reports of thermal sensations in the lower limbs*”).

When asked if he was moving any of his lower limbs, the patient often replied “no” since he was not aware of these movements while wearing the VR headset. His awareness of these movements only occurred when video measurements were taken with the lower limbs exposed or at the end of the decoding stage, which coincided with the conclusion of the sessions when the questionnaires were administered.

These additional manipulations suggest that the presence of lower limb movements may be related to the use of the BCI, the patient engagement, and the absence of stress factors.

### 3.8. Additional Findings

Due to the novelty of our setup, we report here an additional number of findings from our sessions that may or may not be directly related to the intervention applied. They are presented to ensure that (i) they are documented in the scientific literature allowing future studies to confirm or refute any potential relation to similar BCI interventions, and that (ii) health professionals involved in BCI applications can be aware of the potential effects and responses that may occur.

#### 3.8.1. Reports of Thermal Sensations in the Lower Limbs

Throughout the intervention period, the patient reported experiencing thermal and tactile sensations that were unexpected, given his previous experiences and AIS classification of his injury. Specifically, in seven sessions (S13, S15, S21, S23, S28, S29, and S32), he described feeling cold in his lower limbs. These sensations were entirely novel to him within the 32 years following his injury. These sensations predominantly occurred during scenarios in which the avatar walked in water and often coincided with lower limb movement patterns (5/7 = 71.33%). While occasionally described as unpleasant (S15 and S23), these sensations were not associated with pain. The patient’s reports indicated that these sensations did not deter him from continuing to participate in the subsequent sessions.

#### 3.8.2. Patient Engagement

The 14-month period during which this intervention occurred included several significant life events, also referred to as transitions [46], that could have had a substantial impact on the patient’s participation, pain levels, engagement, and overall quality of life. Notably, among these events were (1) two periods of confinement associated with the COVID-19 pandemic; (2) a change in family circumstances; and (3) a shift in macroeconomic conditions. Despite these challenges, the patient’s willingness to continue with the sessions remained undiminished. He consistently expressed, “I always find new things in the scenarios, so I don’t get bored”, indicating a strong desire to continue participating in the study in the future.

When directly asked about the intervention’s effects on his well-being and quality of life (during interviews conducted at the end of each session and in the assessments at the beginning and end of each intervention cycle), the patient consistently expressed his eagerness to continue the sessions even after the study’s conclusion. He also mentioned that “the sensation of standing and walking through the scenarios” provided moments of relaxation and well-being that enhanced his overall quality of life.

## 4. Discussion

The present study examines the effects of extending the application of a BCI-based neurorehabilitation protocol from 1 to 14 months. This protocol utilized recorded brain activity, captured through EEG, to control an avatar’s movements in various hyper-realistic scenarios, providing haptic, thermal, visual, and auditory feedback to the user.

The extended protocol maintained or reduced pain levels throughout the testing period. Changes in the numerical pain scales were correlated with the power of the theta frequency band at the F3 electrode. Comprehensive changes in EEG activity occurred throughout the testing period, suggesting neuroplasticity across an extensive neural network encompassing frontal, central, parietal, temporal, and occipital regions. The continued implementation of this protocol resulted in sessions where alternating lower limb movements appeared, resembling automatic gait patterns. The presence or absence of these movements may be partially attributed to factors such as stress and patient engagement, though the underlying neural mechanisms remain unclear. Furthermore, the use of hyper-realistic scenarios, including walking in water, elicited cold sensations in the lower limbs, which, while unusual, were not painful. These cold sensations did not impede the emergence of lower limb movement patterns. Lastly, the patient’s AIS grade remained unchanged.

### 4.1. Sustained Pain Reduction and Neural Correlates

Over 14 months, the intervention resulted in a sustained reduction in self-reported pain levels using three different pain scales. These self-reported changes in pain were accompanied by changes in neurophysiological activity suggestive of neuroplasticity at frontal electrodes [32]. In a prior study, we reported that the implementation of this intervention over a short period led to reductions in pain levels [10]. In our current study, we found that these reductions were maintained for the entire 14-month duration. It should be noted that clinical assessments a1, a2, and a3 (Figure 3A,B) were not performed on the same days that the motor imagery task was implemented. This means that the self-reported pain levels obtained from these assessments were not the result of the patient being exposed to the VR environment immediately before the evaluation. The patient also expressed a general improvement in his quality of life during the interviews.

The maintenance of reduced pain levels over this extended period holds significant importance, especially when considering multiple variables that could potentially influence these values. Notably, this extended timeframe allows us to eliminate the possibility that the observed results were due to factors like seasonal changes [47,48], novelty effects associated with technology’s implementation, or alterations in medication. Additionally, it is noteworthy that the patient encountered highly stressful events in both personal and work life throughout these 14 months, yet these did not lead to significant increases in pain levels [49].

In addition to the sustained reduction in pain levels, neurophysiological changes were also observed in the power of delta and alpha frequency bands across a network of electrodes recording above the frontal, central, temporal, parietal, and occipital regions [19,32,50]. Furthermore, a negative correlation was also identified between changes in pain levels and theta band power in the sessions of the second part of the study, supporting Hypothesis 1. As neuropathic pain associated with SCI has been described as a thalamocortical dysrhythmia characterized by an increase in power in the lower frequencies [19,32,50,51], it is relevant that the neurophysiological findings described here occurred in the same frequency band.

Previous studies already reported correlations between neural activity and neuropathic pain in SCI cases [19,32], and other conditions [52], often showing an increase in power in frontal and other electrodes. Collectively, these findings suggest the presence of neuroplasticity associated with the sustained reduction in pain levels over the 14-month intervention period. However, the specific role of each of the electrodes and frequency bands remain somewhat unclear.

In summary, the findings related to pain are significant because they support the hypothesis that patient-reported pain levels can be analyzed through a network of electrodes in various regions; indicate that the consistent increase in delta band power at the F3 frontal electrode and the reduction in power in the alpha band could be potential markers of neuroplasticity for this specific intervention (since a trend occurred over multiple sessions); and lastly, provide neurophysiological evidence supporting the intervention’s impact on self-reported pain values.

### 4.2. Induction of Alternating Movements of the Lower Limbs

The utilization of a hyper-realistic VR immersive environment with multimodal feedback seems to have resulted in the emergence of non-spastic, repetitive lower limb movements, supporting Hypothesis 2. Meanwhile, no clear differences in the power of the delta frequency band for electrodes C3 and C4 was found, therefore not supporting our Hypothesis 3. This lack of difference may be an indication that the mechanism involving lower limb movement is not related to this frequency band in these electrodes or may be the result of the small number of sessions analyzed.

A simultaneous video analysis of both lower limbs revealed alternating muscle movements resembling a gait pattern. These patterns were automatic in nature as the patient had no conscious control over their initiation or cessation, was unaware of their occurrence until removing the VR headset, and these were not correlated with the motor imagery task. Typically, automatic gait patterns are governed by central pattern generators (CPGs) [53,54,55]. The rhythmic patterns observed during the implementation of this BCI seem to have a cortical component, as suggested by the apparent modulations associated with task engagement and stressful events. However, as we did not specifically test for neurophysiological parameters that could effectively determine if we had activated a CPG, we can only speculate about the possibility of having activated one in the present study.

The presence of central pattern generators at the spinal cord level has been identified in cats and proposed in humans [33,34,35]. These mechanisms generate highly organized patterns of lower limb activity resembling walking, independent of cortical activation. However, the results obtained in this study seem to support the existence of a cortical component, as movements were more often observed only when the patient began using the multimodal VR equipment. While the patient’s AIS classification remained unchanged throughout the intervention (AIS grade A), these results suggest the possibility of intact fibers allowing cortical activation of these patterns.

Regardless of the origin of these movements, one potentially valuable result for future studies on this type of lower limb movement patterns is the possibility to activate them deliberately. This study suggests that, under suitable conditions (i.e., without distractors or stress factors), performing the motor imagery task in the hyper-realistic VR environment may have resulted in the emergence of these movements in a significant fraction of sessions. However, it should be noted that in session S14, the movements appeared before the patient engaged in the motor imagery task.

While this mechanism is interesting and potentially relevant for future clinical approaches, it raises some questions that this study could not address. For instance, the intervention implemented has the potential to initiate this movement pattern but does not provide a means to interrupt it, which therefore makes these movements desynchronized from the patient’s volitional state. Also, these movements persisted for a few minutes in the laboratory space before naturally subsiding. Given that stress appears to hinder the initiation of these movements, we propose that future studies involving a simultaneous distraction and moderate stress inducer (such as removing the VR headset and placing hands in ice water) to investigate whether such intervention can enable controlled interruption of this movement pattern [56]. Also, neuromodulation of the relevant structures through transcranial and epidural stimulation can further help dissect the network associated with these findings. Lastly, a neurophysiological description of the lower limb activation will be relevant to properly describe the details of the underlying mechanism.

### 4.3. Thermal Sensations

In some sessions involving the water scenario, the patient reported experiencing dysesthesia, describing it as “a sensation of cold that started in the feet and then spread to the legs”. This observation, when considered alongside the lower limb movements, suggests that this SCI may not be complete. Further studies employing imaging approaches could help confirm these findings and elucidate their origin. It is worth noting that while the patient found this sensation unpleasant, even going as far as to request the removal of all equipment during a session to confirm if his feet were indeed cold, these experiences did not diminish his willingness to participate in the study. He also mentioned that he had been pondering this matter for a few days.

### 4.4. Relevant Psychosocial Effects

A somewhat unexpected finding in the current study was the apparent influence of stress and various psychosocial-affective factors on the obtained results. Specifically, the patient noted that the sessions provided a recreational opportunity for him to reconnect with the sensation of standing and always offered new details in the scenarios, keeping his interest alive.

### 4.5. Clinical Results and AIS Scale

An assessment of neurological outcomes resulting from the implementation of the current protocol revealed no changes on the AIS scale. While this scale is undoubtedly valuable, it lacks the capacity to assess certain parameters, such as pain, spasticity, and dysesthesia [57]. This suggests that either the present intervention had effects on unmeasured variables within the AIS scale, or the impact achieved by the intervention was not significant enough to be detected by the variables assessed by that scale. Therefore, future studies should consider incorporating additional clinical measurements and/or questionnaires that can more accurately capture the effects of our intervention. For example, it would be important to determine if this lesion can be classified as “discomplete” [58], namely through electroneurography, electromyography, sympathetic skin response, and evoked potentials [59].

Another topic that must be considered here is the issue of evaluating pain and other functions under conditions of spontaneity and stimulation with the expectation of a response [60]. While the assessment with the AIS scale employs short and targeted stimuli (e.g., pin prick), the intervention used in the present study evaluates responses that occur at the neurophysiological and muscular level, as well as self-reports of pain when the user is stimulated in a relatively constant way through his interaction with the VR environment. Therefore, it is both useful and relevant to continue using the AIS scale to evaluate potential effects that may arise from the intervention conducted here. However, the current results of pain evaluation suggest this intervention’s effects may manifest in other responses and physiological mechanisms.

### 4.6. Technical Aspects

The present study was conducted on a patient classified as having a complete spinal cord injury, meaning that the emergence of lower limb movements was not anticipated. It will be crucial to implement this protocol in a larger number of patients with similar and other injuries to determine the short-term, medium-term, and long-term effects, as well as to corroborate the clinical results and neurophysiological mechanisms proposed here.

## 5. Conclusions

The application of a neurorehabilitation protocol based on a multimodal BCI to a patient with an SCI at T4, classified as AIS grade A (complete), resulted in a sustained reduction in pain levels. This reduction was accompanied by neurophysiological changes suggestive of neuroplasticity and the emergence of movements resembling patterns of gait in the lower limbs. While the neuronal mechanisms underlying this observation remain unknown, it appears to be related to stress and engagement, suggesting the existence of a cortical component. It cannot be ascertained from the present data if this mechanism is related to a central pattern generator. In summary, this study suggests that the continued application of this protocol can lead to sustained reduction in pain, potentially improve other clinical variables, and increase reported quality of life.

## Figures and Tables

**Table 1:** Stressful events and absence of lower limb movements. Summary of the events that took place during the initial (1–10) and final (11–34) sessions. In the column “Limb Mov.” are registered the sessions where rhythmic lower limb movements were observed during the implementation of our BCI protocol, given that “L” represents the appearance of left lower limb movements, “R” the right lower limb movements, and “µ” denotes the appearance of micro-movements (which were only evaluated in the last four sessions). In the remaining columns are presented other recorded variables and findings, along with additional notes took during each session. Lower limb movements were present in most sessions where no stressful events occurred and the patient was engaged in the task (i.e., not worried about other problems). N/A: not applicable.

	Session	Limb Mov.	Stress	Engagement	Cold Feet	Additional Notes
Initial (previous study)	1	No	No	Yes	No	N/A
	2	No	No	Yes	No	N/A
	3	No	No	Yes	No	N/A
	4	No	No	Yes	No	N/A
	5	No	No	Yes	No	N/A
	6	No	No	Yes	No	N/A
	7	No	No	Yes	No	N/A
	8	No	No	Yes	No	N/A
	9	Yes: R	No	Yes	No	N/A
	10	Yes: L	No	Yes	No	N/A
Final (present study)	11	No	Yes	Yes	No	Arrived late. Dry mouth.
	12	No	No	Yes	No	Good news at work. Reported less pain.
	13	Yes: L	No/yes	Yes	Yes	Reported cold feet. Session interrupted to drink water.
	14	Yes: L	No	Yes	No	Reported lower pain levels at home. Movement started before the session. Reported having used a different mental strategy.
	15	No, then L (see notes)	Yes	Yes	Yes	Arrived very late and was slightly anxious. After the session, was placed in water scenario. Reported cold feet. Lower limb movements started.
	16	No	Yes	Yes	No	Anxious. Reported problems sleeping. Technical problems.
	17	No	Yes	No	No	Technical problems.
	18	Yes: L	No/yes	Yes	No	Light choking (saliva). Session interrupted.
	19	No	Yes	Yes	No	Moderate choking (saliva). Session interrupted. Participant taken to the emergency room for evaluation.
	20	Yes: L?R?	No	Yes	No	Movement started as soon as the participant entered the VR environment. Slightly anxious about the possibility of choking on saliva. Movement side not annotated in session notes.
	21	Yes: R, L/R, R	No	Yes	Yes	No change in scenario nor session interrupted due to cold feet (not uncomfortable). Right limb movement, but once presented alternated movement in both limbs.
	22	Yes: L	No	Yes	No	Movement amplitude increased throughout session.
	23	Yes: R	No/yes?	Yes	Yes	Movement amplitude increased throughout the session. Became uncomfortable with cold feet.
	24	Yes: L	No	Yes	No	Movements starting during acquisition phase.
	25	Yes: R	No	Yes	No	Small technical problem.
	26	No	Yes	Yes	No	Technical problems. Light choking (saliva). Session interrupted.
	27	No	Yes	Yes	No	Arrived very late. Light choking (saliva). Session interrupted.
	28	Yes: L	No	Yes	Yes	Reported cold feet. Not anxious about the possibility of chocking on saliva.
	29	No	Yes	No/yes	Yes	EEG failed. Instructed initially to not use motor imagery (no movement). Then, instructed to use motor imagery (reported cold feet, but no movements occurred).
	30	Yes: R	No	Yes	No	N/A [file with session notes lost]
	31	Yes: L/µR	No	Yes	No	Small technical problem. Left lower limb with micro-movements on the right.
	32	Yes: µR	No	Yes	No	Micro-movements on the right lower limb.
	33	No	Yes?	No?	Yes	Reported cold feet. Was worried about travelling on the following day.
	34	Yes: L/µR	No/yes?	No?	No	Left limb macro-movements with amplitude increasing throughout the session. Micro-movements on the right lower limb. Halfway reported that sweat was distracting him from the task (30 °C outside).

## Data Availability

The datasets presented in this article are not available to prevent the patient’s identification. Requests for accessing the datasets should be directed to the corresponding author (M.P.-V.).

## Supplementary Materials

The following are available online at https://www.mdpi.com/article/10.3390/life14030396/s1, Video S1: Pattern of alternating movements in lower limbs.
